# Structural and dynamics evidence for scaffold asymmetric flexibility of the human transthyretin tetramer

**DOI:** 10.1371/journal.pone.0187716

**Published:** 2017-12-14

**Authors:** Giuseppe Zanotti, Francesca Vallese, Alberto Ferrari, Ilaria Menozzi, Tadeo E. Saldaño, Paola Berto, Sebastian Fernandez-Alberti, Rodolfo Berni

**Affiliations:** 1 Department of Biomedical Sciences, University of Padua, Padua, Italy; 2 Department of Chemical Sciences, Life Sciences and Environmental Sustainability, University of Parma, Parma, Italy; 3 Universidad Nacional de Quilmes/CONICET, Bernal, Argentina; Griffith University, AUSTRALIA

## Abstract

The molecular symmetry of multimeric proteins is generally determined by using X-ray diffraction techniques, so that the basic question as to whether this symmetry is perfectly preserved for the same protein in solution remains open. In this work, human transthyretin (TTR), a homotetrameric plasma transport protein with two binding sites for the thyroid hormone thyroxine (T4), is considered as a case study. Based on the crystal structure of the TTR tetramer, a hypothetical D2 symmetry is inferred for the protein in solution, whose functional behavior reveals the presence of two markedly different K_d_ values for the two T4 binding sites. The latter property has been ascribed to an as yet uncharacterized negative binding cooperativity. A triple mutant form of human TTR (F87M/L110M/S117E TTR), which is monomeric in solution, crystallizes as a tetrameric protein and its structure has been determined. The exam of this and several other crystal forms of human TTR suggests that the TTR scaffold possesses a significant structural flexibility. In addition, TTR tetramer dynamics simulated using normal modes analysis exposes asymmetric vibrational patterns on both dimers and thermal fluctuations reveal small differences in size and flexibility for ligand cavities at each dimer-dimer interface. Such small structural differences between monomers can lead to significant functional differences on the TTR tetramer dynamics, a feature that may explain the functional heterogeneity of the T4 binding sites, which is partially overshadowed by the crystal state.

## Introduction

Human transthyretin (TTR) is a homotetrameric protein involved in the transport in extracellular fluids of thyroxine (T4) and in the co-transport of vitamin A, by forming a macromolecular complex with plasma retinol-binding protein [1,2]. Its structure was determined in the late seventies and is now known at high resolution [3,4]. The TTR monomer is composed of two four-stranded anti-parallel β-sheets and a short α-helix; two monomers are held together to form a very stable dimer through a net of H-bond interactions involving the two edge β-strands H and F, in such a way that a pseudo-continuous eight-stranded β-sandwich is generated, in which H and F β-strands from each monomer in the dimer are connected to each other by main-chain H-bonds and H-bonded water molecules. Structurally, the TTR tetramer is a dimer of dimers, in which the two dimers associate, interacting mostly through hydrophobic contacts between residues of the AB and GH loops. The assembly of the four identical subunits in TTR is highly symmetrical, being characterized by 222 symmetry. A long channel, coincident with one of the 2-fold symmetry axes, transverses the whole protein and harbors two T4 binding sites at the dimer-dimer interface.

Despite the presence in the TTR tetramer of two identical binding sites, which are both occupied in the crystal with roughly similar mode of binding by T4 [1], its binding in solution is characterized by a strong negative cooperativity, with about two order of magnitude difference in the K_d_ values for the first and second T4 bound to TTR [5]. Recently, additional evidence for TTR binding site heterogeneity both in solution, using the polyphenol resveratrol as a fluorescent ligand [6], and in the crystal [7], has been obtained. More than 240 crystal structures of TTR in complex with a variety of chemically different ligands, whose binding often exhibits negative cooperativity, are present to date in the Protein Data Bank. Nevertheless, the molecular basis of the cooperative behavior and of the heterogeneity of T4 binding sites remains to be clarified.

Human TTR and a number of its mutant forms have been associated with amyloid diseases [8]. Amyloidoses are generated by the misfolding, misassembly and pathological aggregation of several proteins, among which human TTR represents a remarkable example. Evidence has been obtained by JW Kelly and coworkers to indicate that the rate-limiting dissociation of the native tetrameric state into monomers, followed by misfolding of TTR monomers and their downhill polymerization, leads to the formation of protein aggregates *in vitro*, and presumably *in vivo* ([9], and references therein). Following these observations, the properties of a large number of TTR ligands have been investigated in prospect of their use as drugs effective in the therapy of TTR amyloidosis. In fact, T4 and other specific TTR ligands are able to stabilize the TTR tetramer and to inhibit protein aggregation by occupying the T4 binding sites and establishing interactions that connect the couple of subunits that form each binding site [9] [10] [11] [12]. Interestingly, it has been inferred that the degree of negative binding cooperativity of a ligand is inversely related to its ability to saturate and stabilize the TTR tetramer, so that features related to binding cooperativity may also be relevant with regard to the anti-amyloidogenic potential of ligands [12].

Consistent with the observation that monomeric TTR may represent a key species along the pathway of TTR amyloidogenesis, two mutations (F87M-L110M) able to induce the dissociation of TTR into monomers were found to drastically accelerate protein aggregation *in vitro* [13]. An additional mutation (S117E) has been introduced here in the sequence of the double TTR mutant, to obtain a triple mutant, which is characterized by a stronger tendency to dissociate into the monomeric state in solution, in comparison with the double mutant. However, crystal packing in the presence of high protein concentration led to the formation of the TTR tetramer, whose structure has been determined. Here, we report on the comparison of structural features of the triple F87M/L110M/S117E TTR mutant and of other, previously characterized, forms of human TTR, both wild type and mutant forms, crystallized in different space groups. Our data provide evidence for a significant structural flexibility and asymmetric dynamics of the scaffold of the TTR tetramer, a feature that leads to asymmetric functional properties of this protein in solution, such as those associated with its putative cooperative behavior.

## Materials and methods

### Crystallization and structure determination

Recombinant mutant forms (F87M/L110M and F87M/L110M/S117E) of human TTR were prepared by site-directed mutagenesis essentially as described [14]. Crystals of the triple (F87M/L110M/S117E) TTR mutant were grown using the hanging-drop vapor diffusion method. 2 μl of protein (7.3 mg/ml) solution in 50 mM Tris-HCl (pH 8.0), 1 M ammonium sulfate, were equilibrated against a well solution (100 μl) containing 0.1 M sodium phosphate (pH 7.5), 2.2 M ammonium sulfate. Single crystals of approximate size 0.02 mm in the longest dimension were obtained in about a week of incubation at room temperature. 1500 images with an oscillation of 0.15° each were collected at the ID30B beamline of European Synchrotron Radiation Facility (ESRF, Grenoble, France) for a total exposure time of 55.5 s. The crystal belongs to the space group I222, with one monomer in the asymmetric unit. Datasets were processed with the software XDS [15] and scaled with Scala [16] contained in the CCP4 suite [17]. The space group is I222, with one monomer per asymmetric unit (V_M_ = 2.05, estimated solvent content 40%). The physiological tetramer is generated through the crystallographic two-fold axes. The structure was solved by molecular replacement using as a template one monomer of wild-type TTR in the P2_1_2_1_2 space group (PDB ID 4WO0, [7]) and refined using the package Phenix [18]. In the last cycles, TLS refinement was applied. Map visualization and manual adjustment of the models were performed using the Coot graphic interface [19]. Statistics on data collection and refinement are reported in Table 1.

**Table 1:** Data collection and refinement statistics.

Data set	TTR I222
Wavelength (Å)	0.973186
Cell dimensions ***a***, ***b***, ***c*** (Å)	42.3 67.0 83.6
Resolution (Å)	52.29–1.94 (2.01–1.94)*
Reflections (unique)	8849 (687)
*R*_merge_	0.073 (0.916)
*R*_pim_	0.030 (0.514)
*<I* /σ(*I)>*	13.0 (1.6)
*<CC(1*/2)*>*	0.998 (0.396)
Completeness (%)	97.4 (80.5)
Redundancy	7.2 (4.8)
**Refinement**	
No. reflections	8841
*R*_work_ / *R*_free_	0.2296 (0.310) / 0.2671(0.347)
No. protein / solvent atoms	896 / 25
R.m.s. deviations	
Bond lengths (Å)	0.008
Bond angles (°)	0.944
Ramachandran plot	
Favored /outliers (%)	96.5 / 0.0
Rotamer outliers (%) / Cβ- outliers	2.1 / 0
Overall MolProbity score [20]	1.54

*Numbers in parentheses refer to the last resolution shell.

### Normal modes analysis

Normal mode analysis has been calculated using the Elastic Network Model (ENM) [21] [22] [23] [24] [25]. The model represents a protein structure as a network of *N* nodes. Herein, we have considered as nodes the atoms of protein backbone, C_β_ and the center of mass of side chains. Springs connect each node to their neighbors within a cut-off distance *r*_*c*_ = 7Å. The resultant potential energy is defined, according to [21] [26] [27], as

$$E(r_{i},r_{j})=\frac{1}{2}k_{ij}{\left(\left|r_{ij}|-|r_{ij}^{0}\right|\right)}^{2}$$

where ***r***_*ij*_ ≡ ***r***_*i*_−***r***_*j*_ is the vector connecting nodes *i* and *j*, and the zero superscript indicates the position at the crystallographic structure. The value of the force constant *k*_*ij*_ varies according to the type of interaction between nodes *i* and *j* [28] [29]. Normal modes are obtained as a set of eigenvectors {**Q**_**i**_}_i = 1, 3*N*_ of the Hessian matrix, defined as the matrix of second-order partial derivatives of the potential energy. Each **Q**_**i**_ is a 3N vector whose elements {$c_{i}^{j}$}_*j* = 1, 3*N*_ represent the relative displacements of Cartesian coordinates of each *j*^th^ residue. Therefore, for each normal mode **Q**_**i**_, the fraction of relative displacements of residues belonging to subunit A-A’ can be calculated as $\sum_{j\in A-A^{\prime }}{\left(c_{i}^{j}\right)}^{2}$.

### Set of structures representing thermal fluctuations

A set of 1000 structures representing thermal distortions has been generated from the original X-ray (PDB ID 1F41) uncomplexed TTR structure by randomly displacements in the direction of each normal modes *i* within the range [-*A*_*i*_:*A*_*i*_], being *A*_*i*_ (Å) the corresponding amplitude of the mode at room temperature

$$A_{i}={\left(\frac{2k_{B}T}{\lambda_{i}}\right)}^{1/2}$$

where *k*_*B*_ is the Boltzmann constant and *T* is the absolute temperature (300K). *λ*_*i*_ corresponds to the eigenvalue associated to the *i*^*th*^ normal mode scaled in order to best fit the theoretical residue fluctuations with the corresponding experimental temperature factors. The average root mean square difference between structures was ∼ 0.4. The distributions of the fraction of relative displacements of Cα atoms and ligand cavity volumes were evaluated and density histograms with kernel smoothing computed using the R package ggplot2 [30] and software RStudio [31]. The density histogram shows the grain density estimate, which is a smoothed version of the histogram.

## Results

### Crystal structure of the F87M/L110M/S117E TTR mutant form

**Fig 1:**
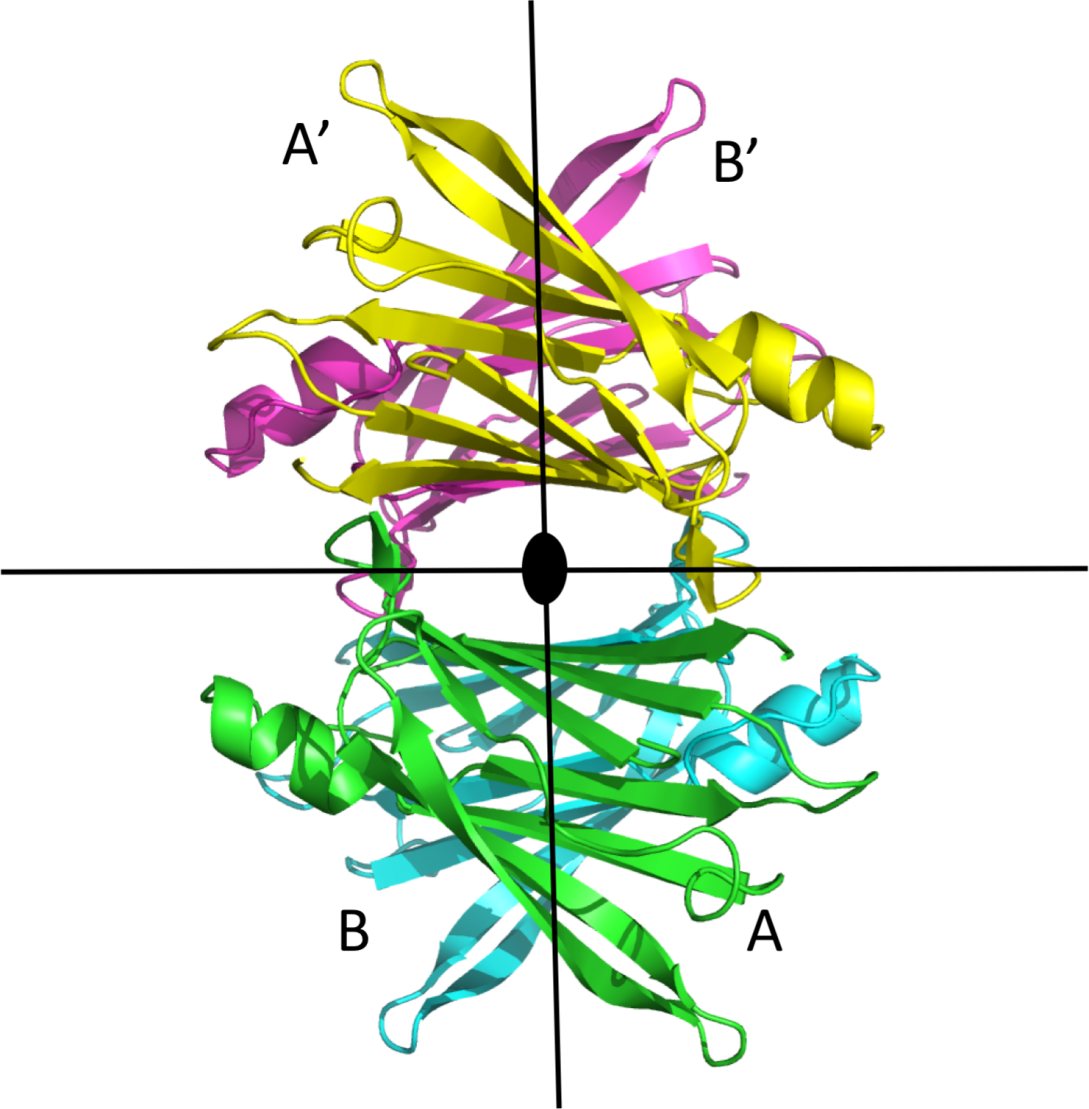
Cartoon view of the TTR tetramer. The two black lines on the plane of the page and the black dot in the center correspond to molecular two-fold axes. In the case of the P2_1_2_1_2 space group, the central dot corresponds to the crystallographic two-fold axis, perpendicular to the plane of the page. In the I222 space group, all three axes are crystallographic elements of symmetry. Chains are all identical, but they are labelled A and B or and A, B, C and D when a dimer or a tetramer is present in the asymmetric unit, respectively.

Out of a total of 240 human TTR structures present in the Protein Data Bank, 218 structures, including those of several TTR mutant forms and TTR-ligand complexes, belong to the orthorhombic space group P2_1_2_1_2. In such structures a dimer is present in the asymmetric unit, and the second dimer is generated by symmetry, owing to the two-fold crystallographic axis coincident with the central channel in the TTR tetramer. The resulting tetramer present in such crystal can deviate from the ideal 222 symmetry, owing to the fact that only one of the two-fold axes is coincident with the crystallographic one. On the contrary, crystals of the structure presented here for the triple F87M/L110M/S117E TTR mutant belong to space group I222, where only one monomer is present in the asymmetric unit, and the tetramer is generated by the crystallographic symmetry (**Fig 1)**. At variance with the structures obtained from crystals belonging to the space group P2_1_2_1_2, in the centered I222 space group, the molecular symmetry of the protein is fully coincident with the crystallographic one. The other known structure in this crystal form is that of the V122I TTR mutant in complex with tolcapone [12]. In both cases the tetramer generated by the crystallographic axes is equivalent to that of the already known structure of TTR [4].

The final model in the I222 space group is essentially the same observed in the case of the P2_1_2_1_2 crystal form. In fact, the r.m.s.d. for the superposition of 114 equivalent Cα atoms of the monomer of the triple F87M/L110M/S117E TTR mutant with those of a representative wt TTR structure (PDB 1F41 [4] is 0.52 Å for monomer A and 0.78 Å for monomer B. Similar low r.m.s.d. for the superposition of the wt TTR structure (PDB 1F41) to TTR crystallized in other space groups are also found: 0.39 Å for the V122I TTR mutant in complex with tolcapone (PDB 5A6I [12]); 0.45Å for the double F87M-L110M TTR mutant (PDB 1GKO [13]); 0.60 Å for wt TTR in complex with 4-hydroxy-chalcone (PDB 5EZP [32]); 0.74 Å for the monoclinic C2 crystals of the L55P TTR mutant (PDB 5TTR [33]); 0.64 Å for the wt TTR monoclinic P2_1_ crystals (PDB 1ICT [34]).

The triple F87M/L110M/S117E TTR mutant in solution is characterized by a high propensity to keep a monomeric state in solution, greater than that of the double F87M/L110M TTR mutant, even in the presence of the strong fibrillogenesis inhibitor tafamidis [10] (**S1 Fig**). The main reason for the pronounced tetramer destabilization could be due to the presence of the side chains of two pairs of Glu117, one towards the other, in the inner part of the cavity for each couple of subunits (A-A’ and B-B’). The distances between the two Oε1 and Oε2 of Glu117 residues of subunits A and A’ are in fact 5.15 Å and 5.06 Å, respectively, thereby generating a strong electrostatic repulsion, provided that they are negatively charged. On the other hand, the distance between two Oε2 atoms of Glu117 of subunits A and B’ (and of B and A’) is 2.79 Å in the crystal, which is consistent with the formation of H bond interactions between each couple of the above subunits and, consequently, with the presence of tetrameric TTR in the crystal. The different aggregation state found for the protein in the crystal and in solution may depend on contacts between subunits and dimers induced by crystal lattice constraints and on differences in pKa values of the carboxylic groups of Glu117 residues of the proteins in the two physical states.

### Relationships between monomers for different TTR crystal forms

To analyze the structural differences induced by the presence or absence of the crystallographic symmetry for structures determined from crystals belonging to different space groups, we have compared several TTR structures, as follows: the triple F87M/L110M/S117E TTR mutant; the wild type TTR form (PDB 1F41 [4]), as representative of a high-resolution structure of wild type TTR; the double F87M/L110M TTR mutant, which crystallizes in the P2_1_2_1_2_1_ space group with a tetramer in the asymmetric unit (PDB 1GKO, [13]); the V122I TTR mutant in complex with tolcapone (PDB 5A6I, [12]), the only other TTR structure containing a single monomer in the asymmetric unit; the wild type TTR in complex with 4-hydroxy-chalcone (PDB 5EZP, [32]), which crystallizes in the P3_1_ space group, with two tetramers in the asymmetric unit. In the latter case, only one tetramer was considered in the comparison. Data for the structure of the L55P TTR mutant (PDB 5TTR, [33]), crystallized in space group C2 with one tetramer and two dimers in the asymmetric unit, are not reported in detail, but the general behavior is the same, as established for the other TTR crystal forms.

**Fig 2:**
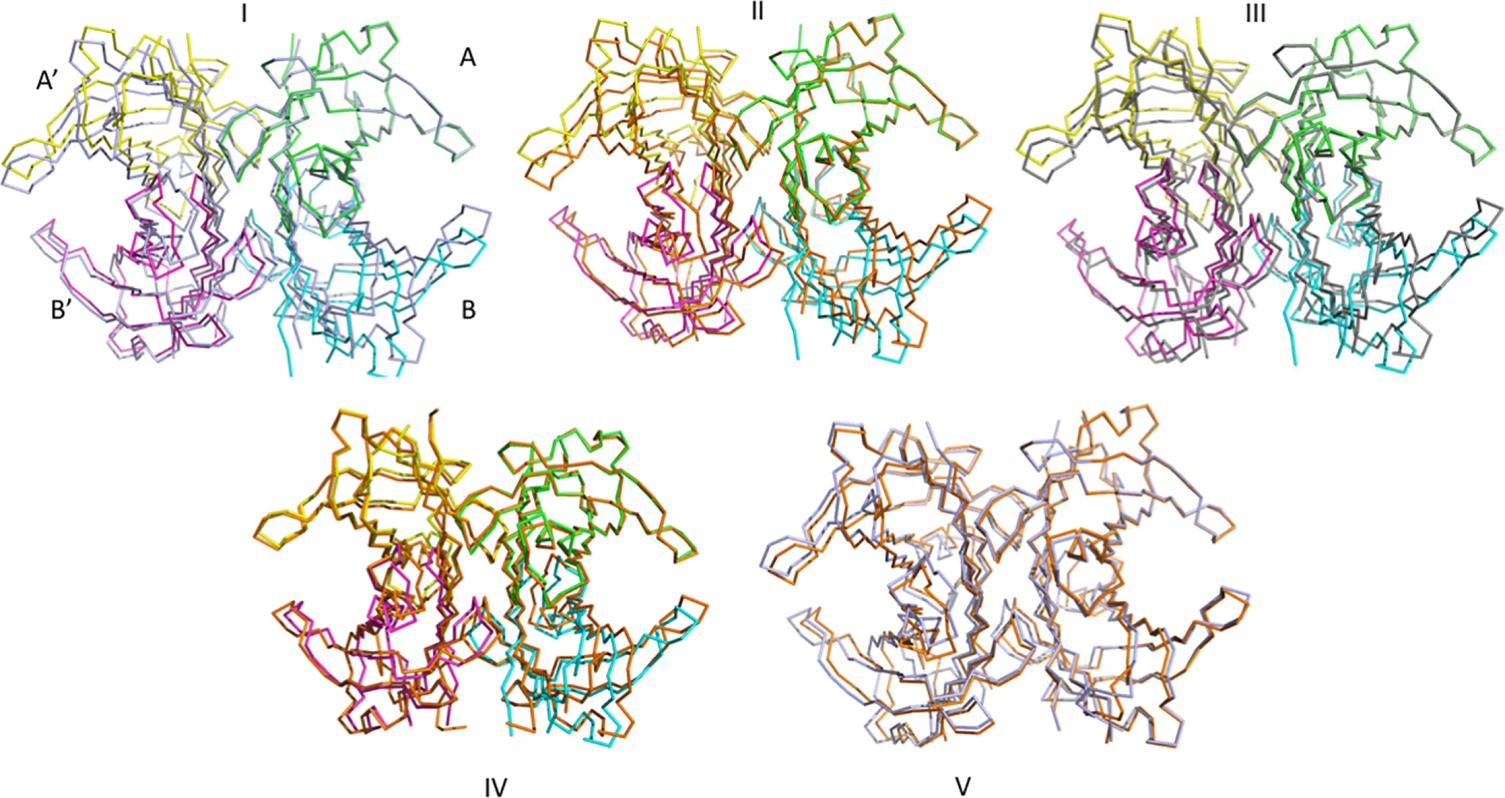
Comparison of the structures of TTR from different crystal forms. Superposition of Cα chain traces of (I) triple F87M/L110M/S117E TTR mutant to 1F41 structure, (II) triple TTR mutant to 56A1 structure, (III) triple TTR mutant to double TTR mutant 1GKO structure, (IV) triple TTR mutant to 5EZP structure, (V) 1F41 to 56A1 structures. In all cases, only monomers A were superimposed. The four monomers of the TTR triple mutant are shown in different colors, the others in the same color.

If the Cα atoms of one subunit, say A, are superimposed, we can visualize the differences in the position of the other subunits in relationships with that of subunit A for different crystal structures/space groups (**Fig 2**). In Table 2, a more quantitative estimate of the differences is given by the measure of the distances between equivalent Cα atoms for subunits B, A’ and B’. An analysis of these distances indicates that by superimposing monomers A of TTR tetramers from crystals belonging to different space groups, monomers B, A’ and B’ are displaced apparently in a random way. This indicates that taking monomer A as reference, the other monomers present a slightly different orientation for different crystal forms. For example, with the crystallographic two-fold axis of space group P2_1_2_1_2 running vertical in the page, by comparing the structures of the triple F87M/L110M/S117E TTR mutant and of wild type TTR (PDB 1F41), monomers B’ superimpose quite well, whilst B and A’ are significantly displaced (**Fig 2, panel I**). On the contrary, in the superposition of 1F41 and 5A6I structures A and B are nearly coincident, while the positions of A’ and B’ diverge significantly (**Fig 2, panel IV**).

**Table 2:** Interatomic distances between equivalent atoms in different TTR tetramers.

	87/110/117 TTR mutant–wild type TTR (1F41)	87/110/117 TTR mutant—V122I TTR mutant (5A6I)	87/110/117 TTR mutant—87/110 TTR mutant (1GKO)	1F41 wild typeTTR—V122I TTR mutant (5A6I)	87/110/117 TTR mutant—4-hydroxy-chalcone—TTR complex(5EZP)
Thr 96 B	2.44	1.71	2.02	0.98	2.37
Thr 96 C (A’)	1.29	1.97	2.08	1.73	1.15
Thr 96 D (B’)	2.38	1.42	2.58	2.69	2.36
Leu55 B	1.77	2.26	2.13	0.77	1.06
Leu55 C (A’)	1.86	1.56	1.45	0.47	0.80
Leu55 D (B’)	2.27	1.52	2.67	2.11	1.90
Ser85 B	3.52	0.96	0.99	3.03	2.37
Ser85 C (A’)	3.42	2.39	3.27	2.65	2.26
Ser85 D (B’)	3.98	2.83	2.48	1.81	2.68

Distances (in Å) between Cα atoms for pair of proteins in subunits B, C and D, after superimposing subunit A of the models. Residues of monomer A are not indicated, since they are practically coincident. C and D labels correspond to A’ and B’ in the P2_1_2_1_2 space group, i.e. the crystallographic two-fold axis superimposes A’ to A and B’ to B.

In turn, this situation has consequences on the size of TTR binding cavities. To give an indication of the size of each of the two cavities, distances between corresponding Cα atoms of monomers A–A’ and B–B’ (i.e. the couples of subunits that line the two T4 binding cavities) are compared in **Table 3**. Interestingly, these distances are in some cases quite different from one structure to the other, a fact possibly due to real differences in the size of the cavity (also considering that two of the reported structures are those of TTR mutant forms). However, such differences could also partially reflect the slightly different cell parameters of the structures considered. More relevant, since not affected by systematic errors, is the internal comparison between the same distance between residues in the cavities formed by monomers A–A’ and B–B’. When only a TTR monomer is present in the asymmetric unit, i.e. a perfect tetramer is present in the crystal, the two cavities are identical by symmetry; in the other cases, where a dimer or an entire tetramer is present in the asymmetric unit, the two may differ in size. As expected, distances between residues close to the center of the tetramer are less affected by the rotation of one monomer relative to the other, whilst those far from the center of the tetramer present larger differences. These differences are very small for wild type TTR (PDB 1F41), in which one dimer is present in the crystal asymmetric unit, and definitely larger in cases where an entire tetramer is present in the asymmetric unit, as for the 4-hydroxy-chalcone in complex with TTR and for the double F87M/L110M TTR mutant. In the latter, the most astonishing difference is represented by residues T119, for which there are more than 4Å differences in the distances between A—A’ and B—B’ (the latter are labeled A–C and B–D in the original structure, since there is a tetramer in the asymmetric unit). It must be considered anyhow that all the examined structures have been determined at different resolutions.

**Table 3:** Distances (in Å) between Cα atoms of subunits A and C (or A’) and B and D (or B’).

	87/110/117 TTR mutant A–A’	87/110 TTR mutant (1GKO) A–A’ / B–B’	V122I TTR mutant (5A6I) A–A’	wild type TTR (1F41) A–A’ / B–B’	4-hydroxy-chalcone—TTR complex (5EZP) A–A’ / B–B’
S(E)117	9.67	9.54 / 9.92	8.75	9.36 / 9.30	9.83 / 9.86
T119	14.17	15.19 / 11.63	13.45	13.30 / 13.17	13.47 / 13.77
A108	11.70	10.45 / 11.82	11.98	11.84 / 11.86	11.56 / 11.73
K15	13.81	12.65 / 14.57	14.14	13.85 / 13.88	13.63 / 13.93
T106	17.72	17.82 / 16.27	17.59	17.94 / 17.80	17.84 / 18.30

In the case of the presence of a perfect tetramer in the asymmetric unit only one distance is reported.

### Normal mode analysis of the TTR tetramer

**Fig 3:**
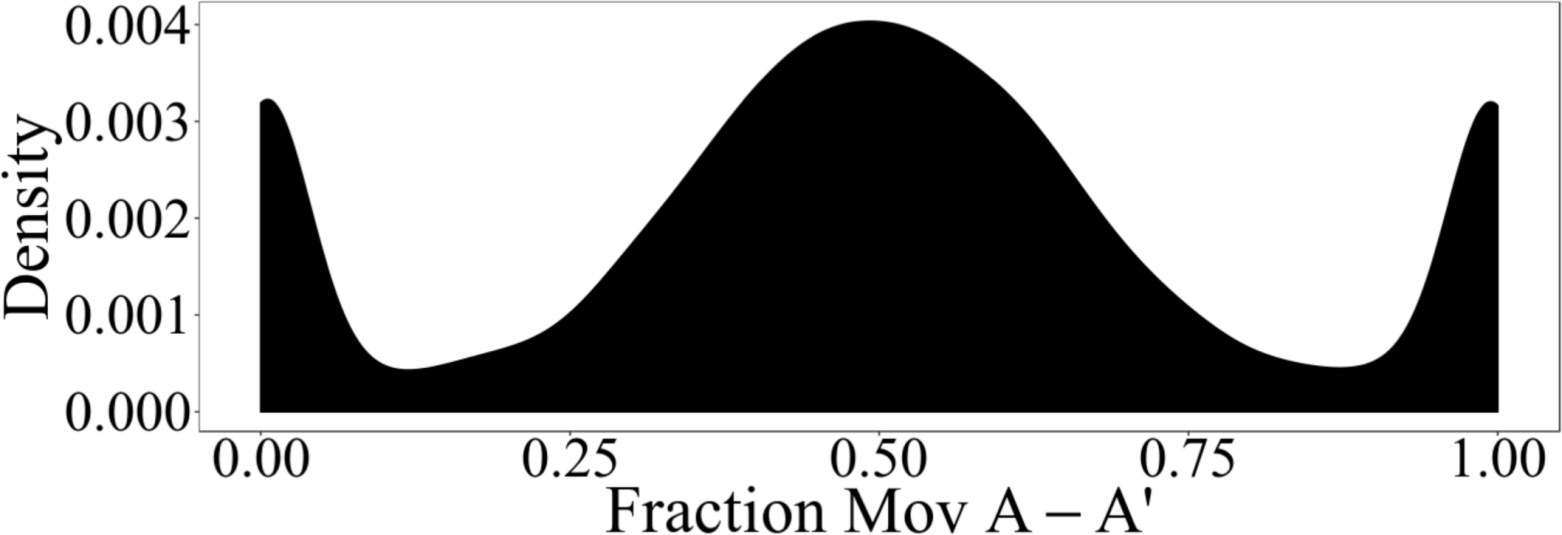
Displacement of subunits. The fraction of relative displacements involving Cα atoms of subunit A-A’ in the ligand-free wild-type TTR tetramer was evaluated and the frequency of appearance in all normal modes is shown as a kernel density plot.

Using normal mode analysis, we have analyzed differences in the flexibilities of residues in the couples of subunits A-A’ and B-B’, which form the two binding sites at the dimer-dimer interface in the TTR tetramer. For this purpose, the fraction of relative displacements involving Cα atoms of subunit A-A’ has been calculated for each normal mode of the wild type ligand-free TTR tetramer (PDB 1F41). The distribution of these values is depicted in **Fig 3.** The peak at values of ∼1 corresponds to normal modes entirely localized on the A-A’ moiety, while normal modes localized on the B-B’ moiety are represented by the peak at ∼0. The maximum at ∼0.5 indicates that most of normal modes are equally distributed between both moieties. Nevertheless, the distribution is not completely symmetric.

In order to analyze functional aspects of the structural and dynamics asymmetries between subunits A-A’ and B-B’, the volumes of ligand-binding cavities at each dimer-dimer interface have been calculated for a large number of structures representing thermal distortions of the crystal structure of the wild type ligand-free TTR tetramer (PDB 1F41). Volumes are obtained combining convex hull algorithm [35] and Delaunay triangulations.

**Fig 4:**
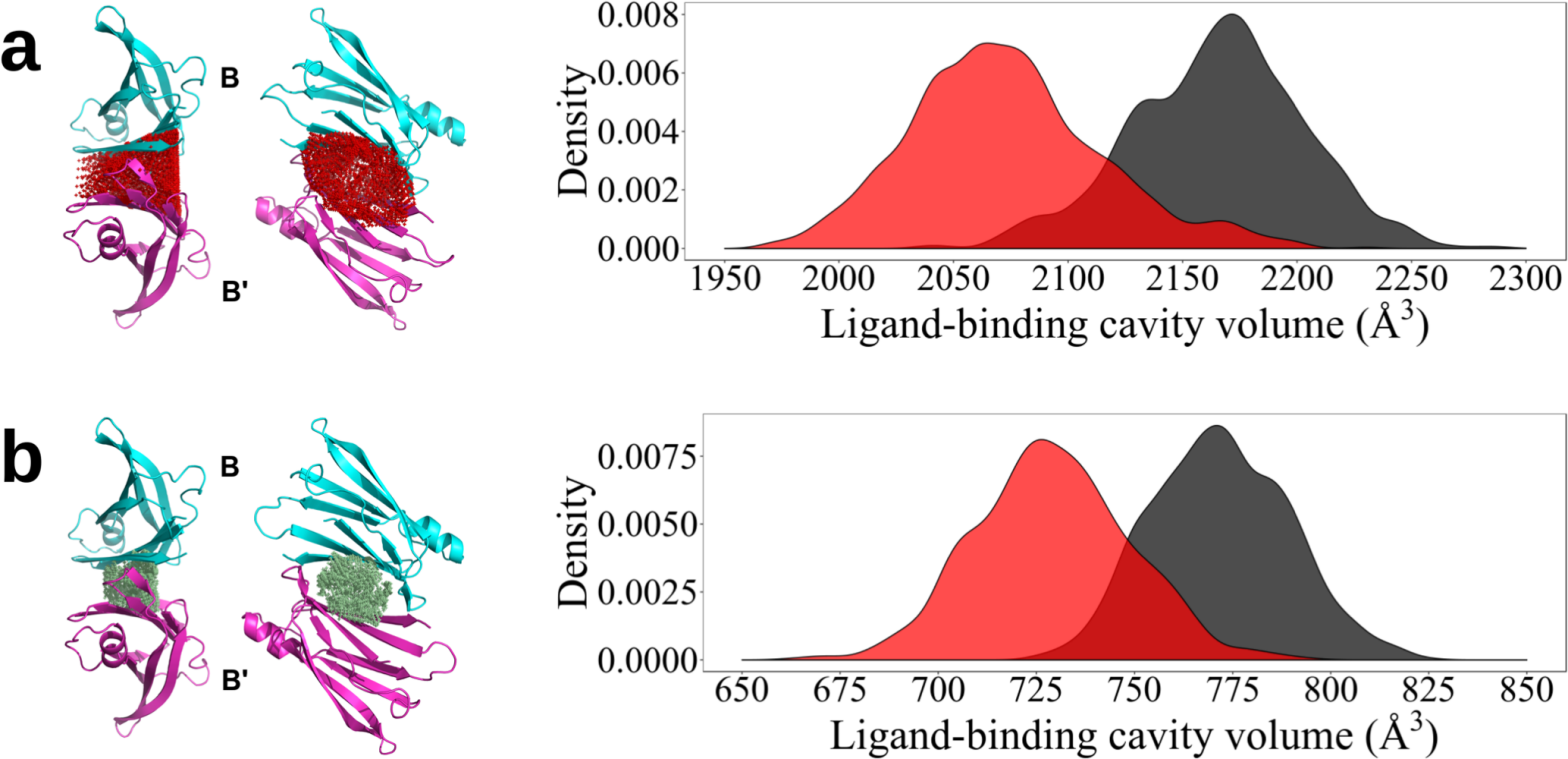
Ligand-binding cavities and their corresponding thermal fluctuations. Ligand cavities are defined according to (a) the 33 residues per subunit and (b) only the 10 buried residues, all listed in **Table 4**. The corresponding distributions of volumes, calculated for a large number of structures representing thermal distortions of the crystal structure of the wild type ligand-free TTR tetramer (PDB 1F41), are depicted as kernel density plots for the cavities either at the A-A’ or at B-B’ (bold) interfaces, respectively.

Ligand-cavities are analyzed either considering all residues per subunit lining the cavities, listed on **Table 4**, or taking into account only the 10 residues that directly interact with a ligand as defined in [36]. **Fig 4** depicts the resulted distribution of ligand-cavity volumes for each of the cavities at the A-A’ and B-B’ interfaces. As can be seen, thermal fluctuations reveal differences in size and flexibility for ligand cavities at each dimer-dimer interface. This is observed for both types of cavities, defined either using all residues lining the cavities or only those residues interacting with the ligand.

**Table 4:** Residues that define TTR ligand-cavity.

LEU 12	GLU 54	LEU 111
MET 13	LEU 55	SER 112
VAL 14	HIS 56	SER 115
**LYS 15**	GLY 57	TYR 116
VAL 16	ARG 104	**SER 117**
**LEU 17**	TYR 105	**THR 118**
ASP 18	**THR 106**	**THR 119**
SER 50	ILE 107	ALA 120
GLU 51	**ALA 108**	**VAL 121**
SER 52	**ALA 109**	VAL 122
GLY 53	**LEU 110**	THR 123

Residues at the halogen binding pocket are denoted in bold.

Here, normal mode analysis has been used to enlighten asymmetric aspects of TTR tetramer dynamics. While most of normal modes are delocalized between subunits A-A’ and B-B’ (**Fig 3**), several modes are mainly localized on one of them. In order to further analyze this finding, TTR-tetramer normal modes have been classified as follows. (1) *symmetric normal modes*: vibrations delocalized between subunits A-A’ and B-B’ with fractions of motions on subunit A-A’ (**Fig 3**) within the range [0.45:0.55] and (2) *asymmetric modes*: modes localized preferentially on one subunit (fraction of motions on subunit A-A’ <0.45 or > 0.55). Modes (2) can be further classified as (2a) *asymmetric modes by differences in relative amplitudes*: modes involving similar motions with different amplitudes on each subunit, (2b) *asymmetric modes by pairs*: modes displaying different motions on each subunit, but with a counterpart mode related to them by 2-fold rotational symmetry, that is, involving equivalent motions but on the other subunit and (2c) *fully asymmetric modes*: asymmetric modes that represent relative displacements on one subunit without a counterpart on the other subunit. Following this classification, we have found that only 18.5%, 1.1% and 16.4% of modes correspond to types (1), (2a) and (2b) respectively, while 64% of modes are fully asymmetric modes (2c).

## Discussion

The molecular symmetry of multimeric proteins is generally determined by using X-ray diffraction techniques, so that the basic question as to whether this symmetry is perfectly preserved for proteins in solution remains open. In this respect, it should be pointed out that the crystal state favors the presence of symmetrical objects, but, at the same time, different crystal contacts and lattice constraints on different parts of the protein could alter its symmetry, introducing small, but significant, deviations from the perfect symmetry. Despite the fact that crystal packing forces can favor a particular sub-state of a protein, in general they are not believed to be strong enough to alter significantly its tertiary and quaternary structures.

In the case of TTR, a tetrameric molecule characterized by three perpendicular two-fold axes, one would expect in solution, where crystal contacts and constraints are absent, an ideal, fully symmetrical tetramer. Subunits that are labeled A and B (and A’ and B’) in the crystal become indistinguishable in solution. On the other hand, the presence of a strong binding heterogeneity for the TTR tetramer in solution suggests that its functional properties are highly affected by conformational changes, allowed by a protein structural flexibility that could not be revealed by X-ray crystallography, a technique that can provide only static structural models trapped in a three-dimensional lattice. Indeed, in a previous work, a molecular dynamics simulation has suggested that in solution the TTR tetramer is quite flexible and that concerted movements affect the relative orientation of subunits [7]. During these structural fluctuations, the two cavities of TTR become larger and smaller in comparison with the theoretical size generated by a perfect 222 symmetry. It was so postulated that the crystallization conditions may select one specific state of the tetramer, perhaps more (or less) symmetrical as compared to that present in solution.

In this work, taking advantage of the crystallization of a TTR mutant form which crystallizes with one single monomer in the asymmetric unit, we have examined and compared in depth the aspects of the symmetry of the TTR tetramer in five different crystal forms, with the presence of a different protein aggregation state in the asymmetric unit. This analysis shows that the orientation of the four monomers relative to each other can change significantly, inducing in such a way some changes in T4 binding cavities. Most importantly, when only one monomer is present in the asymmetric unit and the tetramer is generated by the crystallographic two-fold axes, the perfect symmetry of the tetramer is observed, whilst in the presence of a dimer or of a tetramer in the asymmetric unit a significant deviation from the ideal 222 symmetry is observed.

The results of normal mode analysis are in full agreement with the previous conclusions: they indicate that most of TTR-tetramer vibrations do not present 2-fold rotational symmetry relative to the crystallographic axis that separates subunits A-A’ and B-B’. Moreover, only a few of them represent vibrations that are replicated on both subunits. Therefore, it is expected that these asymmetries on vibrational patterns of subunits A-A’ and B-B’ should be reflected on different dynamical properties relevant for ligand-binding. The asymmetric vibrational patterns for both dimers lead to differential thermal structural distortions and consequent differential functional properties for both ligand cavities.

It is well established that the two binding sites of TTR are characterized by two K_d_ values for most ligands [5] [10] [37], with the second one often being more than one or two orders of magnitude larger in comparison with the first one. A negative cooperativity effect for ligand binding cannot simply be explained on the basis of the several crystal structures of TTR present in the PDB, since in general the two binding sites are very similar and differences, when present, are smaller than the standard deviation of the measurement. This also happens when one of the two binding sites is empty or not fully occupied [7]. Our data strongly support the hypothesis that the two binding cavities of TTR can be different, and that it is the crystallization process that selects a specific conformational sub-state of the tetramer. Accordingly, the flexibility of the tetrameric protein scaffold in solution would permit a dynamic reorientation of subunits, and a consequent repositioning of residues lining the two binding cavities. As a consequence of previously discussed asymmetries in the vibrational patterns of both subunits A-A’ and B-B’, thermal fluctuations leads to differences in size and flexibility for ligand cavities at each dimer-dimer interface (see **Fig 4**). These differences are larger between expanded cavities, defined by all residues at their surface, than between smaller cavities, defined by only those residues interacting with the ligand. Therefore, our results point out to potential differences on either ligand binding and ligand entrance. The binding of a ligand to one of the two cavities, the most favorable one at the moment of binding, possibly freezes the conformation of the tetramer in a slightly asymmetric state, leaving the other binding site in a less favorable conformation for the binding of a second molecule. The second K_d_ is generally larger than the first one, but the binding still takes place, suggesting that the perturbation of the second binding site is relatively small. Owing to the flexibility of the TTR scaffold, the crystallization process could force the tetramer towards a more symmetrical conformation as compared to the state of the protein in solution. This may explain the finding of a rather symmetrical arrangement of the subunits forming the T4 binding site in the TTR tetramer in the crystal, at variance with their remarkable functional heterogeneity in solution.

## Conclusions

It is worth wondering whether the behavior described in this paper is peculiar to TTR, or can be of more general significance for multimeric proteins made by identical subunits and characterized by some kind of rotational symmetry. Based on the crystal structure, it is generally assumed that a perfect symmetry structurally characterizes these proteins in solution, so that a functional symmetry is also inferred. Taking into account that the crystallization process favors the presence of symmetrical molecules in the crystal, and on the basis of the results presented here, the above conclusion could not be always justified.

## Supporting information

S1 Fig Aggregation states for mutant forms (F87M/L110M and F87M/L110M/S117E) of human TTR in solution. Wild type and mutant forms of human TTR, at a concentration of 0.5 mg/ml in 16 **μ**l of 50 mM sodium phosphate, 150 mM sodium chloride, pH 7.5, in the presence (+T) or in the absence (-T) of 30 **μ**M tafamidis (dissolved in DMSO), were analyzed by SDS-PAGE after quaternary structure fixation by incubation with 4 **μ**l of 25% (v/v) glutaraldehyde for 5 minutes at room temperature. The cross-linking reaction was terminated by the addition of 5 **μ**l of sodium borohydrate (7% w/v in 0.1 M NaOH). Samples that were not cross-linked (NCL) were also analyzed for a comparison.
(PDF)
