# Supplementary material for: Structural and dynamics evidence for scaffold asymmetric flexibility of the human transthyretin tetramer
Source: PLoS One. 2017 Dec 14;12(12):e0187716. doi: 10.1371/journal.pone.0187716 (PMC5730205; doi:10.1371/journal.pone.0187716)

# STRUCTURAL AND DYNAMICS EVIDENCE FOR SCAFFOLD ASYMMETRIC FLEXIBILITY OF THE HUMAN TRANSTHYRETIN TETRAMER

Giuseppe Zanotti [1], Francesca Vallese [1], Alberto Ferrari [2], Ilaria Menozzi [2], Tadeo E. Saldaño[3], Paola Berto [1], Sebastian Fernandez-Alberti [3], and Rodolfo Berni [2]

[a] Department of Biomedical Sciences, University of Padua, Padua, Italy
[b] Department of Chemical Sciences, Life Sciences and Environmental Sustainability, University of Parma, Parma, Italy
[c] Universidad Nacional de Quilmes/CONICET, Bernal, Argentina

**Fig. S1. Aggregation states for mutant forms (F87M/L110M and F87M/L110M/S117E) of human TTR in solution.** Wild type and mutant forms of human TTR, at a concentration of 0.5 mg/ml in 16 µl of 50 mM sodium phosphate, 150 mM sodium chloride, pH 7.5, in the presence (+T) or in the absence (-T) of 30 µM tafamidis (dissolved in DMSO), were analyzed by SDS-PAGE after quaternary structure fixation by incubation with 4 µl of 25% (v/v) glutaraldehyde for 5 minutes at room temperature. The cross-linking reaction was terminated by the addition of 5 µl of sodium borohydrate (7% w/v in 0.1 M NaOH). Samples that were not cross-linked (NCL) were also analyzed for a comparison.

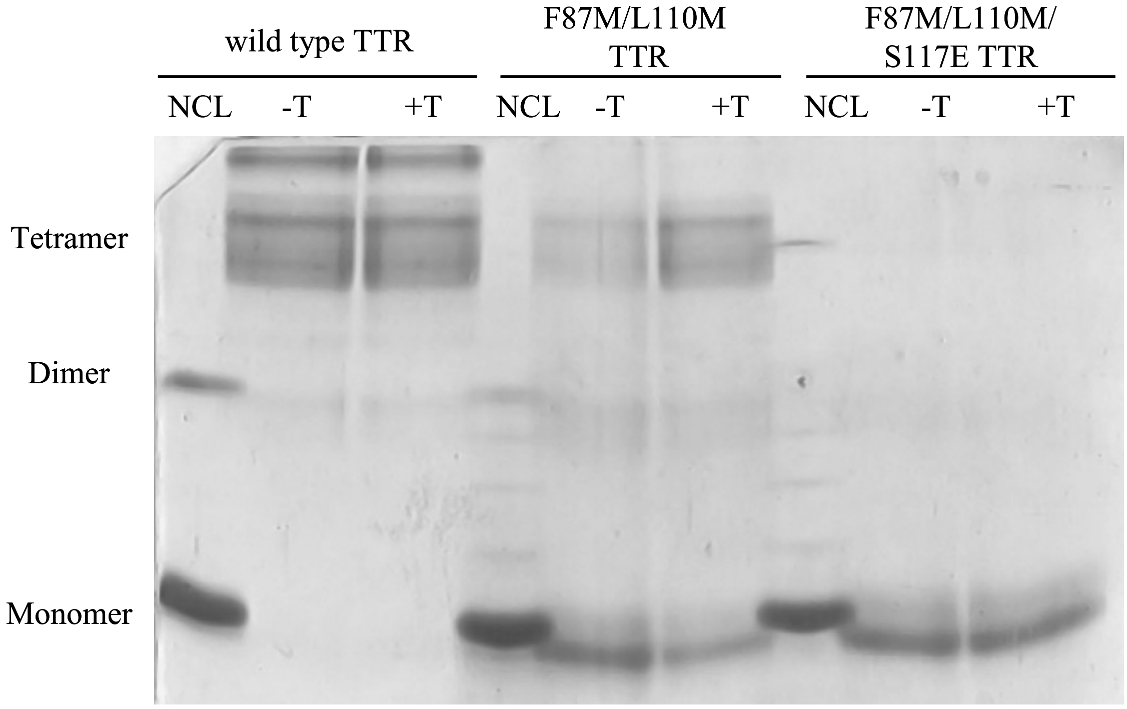

Supplement: S1 Fig — Wild type and mutant forms of human TTR, at a concentration of 0.5 mg/ml in 16 μl of 50 mM sodium phosphate, 150 mM sodium chloride, pH 7.5, in the presence (+T) or in the absence (-T) of 30 μM tafamidis (dissolved in DMSO), were analyzed by SDS-PAGE after quaternary structure fixation by incubation with 4 μl of 25% (v/v) glutaraldehyde for 5 minutes at room temperature. The cross-linking reaction was terminated by the addition of 5 μl of sodium borohydrate (7% w/v in 0.1 M NaOH). Samples that were not cross-linked (NCL) were also analyzed for a comparison. (PDF) [file pone.0187716.s001.pdf]
